# Morphological Structure of the Aortic Wall in Deep Diving Cetacean Species: Evidence for Diving Adaptation

**DOI:** 10.3390/vetsci9080424

**Published:** 2022-08-11

**Authors:** Blanca Mompeó, Simona Sacchini, María del Pino Quintana, Miguel Rivero, Francesco Consoli, Antonio Fernández, Yara Bernaldo de Quirós

**Affiliations:** 1 Department of Morphology, Campus Universitario de San Cristobal, University of Las Palmas de Gran Canaria, 35016 Las Palmas de Gran Canaria, Spain; 2 Veterinary Histology and Pathology, Veterinary School, Institute of Animal Health, University of Las Palmas de Gran Canaria, 35416 Arucas, Spain; 3 Department of Math, Edificio de Informática y Matemáticas, Campus Universitario de Tafira, University of Las Palmas de Gran Canaria, 35017 Las Palmas de Gran Canaria, Spain; 4 Department of Integrative Physiology, University of Colorado Boulder, Boulder, CO 80303, USA

**Keywords:** marine mammal, Odontoceti cardiovascular system, lamellar unit, aortic artery diving habit

## Abstract

**Simple Summary:**

Diving implies cardiovascular adaptations in marine mammals. The work aimed to analyze the aortic wall in nine cetacean species with deep diving habits belonging to four families. We hypothesize that variations in the aortic wall will reflect the diving capability of each species. The analyses showed that the elastic component was higher in the initial part of the artery, the muscular elements increased in the distal portion in all cases, and the apparent organization of the components in the aortic wall did not show essential modifications between the four families. All species presented a decrease in the arterial wall thickness along the aorta. The reduction was dramatic between the initial and thoracic aorta in the sperm whale specimens; meanwhile, the species of the other three families, beaked whale, pygmy sperm whale, and deep-diving dolphins, showed a more uniform decrease between the aortic segments. Because of the diving habits described for the different species, our findings support that a decrease in the arterial wall thickness between the aortic segments is the most relevant characteristic related to diving adaptation.

**Abstract:**

This study analyses the aortic wall structure in nine cetacean species with deep diving habits belonging to four Odontoceti families: Ziphiidae, Kogiidae, Physteridae, and Delphinidae. Samples of ascending, thoracic and abdominal aorta were processed for histological and morphometric studies. The elastic component was higher in the proximal aortic segments, and the muscular elements increased distally in all cases. Morphometric analyses showed that all families presented a decrease in the thickness of the arterial wall and the tunica media along the aorta. The reduction was dramatic between ascending and thoracic aorta in the Physeteridae specimens; meanwhile, the other three families showed a more uniform decrease between the ascending, thoracic and abdominal aorta. The decline was not correlated with a reduced elastic or lamellar unit thickness but with a loss of lamellar units. The organization of the elements in the aortic wall did not show essential modifications between the four families, resembling the structure described previously in the shallow and intermediate diving dolphins. Our findings support that the difference in the morphometric characteristics of the different segments in the aortic wall is likely related to the diving habit more than the absolutes values of any other parameter.

## 1. Introduction

The critical bradycardia and peripheral vasoconstriction during diving suggest a need for cardiovascular system adaptations in marine mammals. Studies on the properties of the aorta in some marine mammals, primarily pinnipeds and some species of toothed whales, showed macroscopic and morphometric differences in the dimensions and structural wall composition in the ascending and other segments of the aorta [1,2,3,4,5]. These characteristics are responsible for mechanical and hemodynamic characteristics of the blood circulation in these animals [6,7,8,9]. The described aortic adaptations in marine mammals for diving included an expansive ascending aorta or an expansive aortic arch and a non-compliant or low compliant descending aorta [2,3,4,5,6] with a greater concentration of elastic fibers in the ascending than in the descending aorta [9,10]. The capacity of the large elastic arteries to store a part of the blood volume with each systole and discharge that volume with the diastole, i.e., the windkessel effect, allows for maximizing the diastolic flow during diving for a more continuous peripheral circulation [11,12]. The morphological features of the aortic wall differ between the species [4,6,9,11]. The wall [IM] thickness, the elastic layer thickness, and the lamellar unit thickness are parameters involved in the compliance and recoil with the blood flow [10]. In the aortic wall of shallow or intermediate diving dolphins, the disposition of the components seems to be similar to that previously described for terrestrial mammals such as pigs, except for a more considerable difference in the proportion of lamellar units between the ascending and thoracic aortic segments [13]. However, the quantity and disposition of those components in the aorta of deep-diving species remains unknown. Considering that deep-diving capabilities imply the existence of adaptations of the cardiovascular system, we hypothesize that morphological and morphometric variation in the aortic wall will reflect the diving capability of each species. Therefore, the study aimed to characterize the structure and morphometry of the aortic wall in some species of four families of Odontoceti with deep diving habits, discuss the obtained data in the four families, and compare them with those observed in other marine and terrestrial mammals.

## 2. Materials and Methods

### 2.1. Animals

This study analyzed the aortic samples from nine deep-diving species belonging to four families: Ziphiidae (*Mesoplodon densirostris* [De Blainville 1817], *Mesoplodon bidens* [Sowerby 1804], *Mesoplodon europaeus* [Gervais 1855], *Ziphius cavirostris* [Cuvier 1823]), Kogiidae (*Kogia breviceps* [De Blainville 1828] and *Kogia sima* [Owen 1866]), Physeteridae (*Physeter macrocephalus* [Linaeaus 1758]), and Delphinidae (*Globicephala macrorhynchus* [Gray 1846] and *Grampus griseus* [Cuvier 1812]). Most of these families are characterized by deep-diving species, except for the Delphinidae family, composed mainly of shallow or intermediate divers. However, *Globicephala macrorhynchus* and *Grampus griseus* are deep divers, so we will refer to them as deep-diving species of the Delphinidae family. Animals studied belonged to different sex and age categories and were stranded along the Canary Islands coasts between 2008 and 2020 (Table 1). Required permission to handle and sample stranded cetaceans was issued by the Environmental Department of Canary Islands’ Government and the Spanish Ministry of Environment. No experiments were performed on live animals. The age categories (calf, young, subadult, or adult) were determined based on body length and sexual maturity [14,15,16]. The categorization of sexual maturity was determined by the morphologic and histologic appearance of the gonads [17]. Regarding diving habits, the deep diver mammals habitually dive deeper than 500 m for foraging, intermediate-divers usually dive between 200–700 m, and shallow-divers descend until 200 m [18].

Necropsies were performed following standardized protocols [19]. The aortas were dissected and processed for histological studies. The ascending aorta was sampled proximally to the arteriosus ligament, the descending thoracic aorta at the middle level of the thoracic cavity, and the abdominal aorta caudal to the mesenteric branch.

### 2.2. Tissue Preparation, Histology, and Histochemistry

Samples were fixed in 10% neutral buffered formalin, dehydrated through graded alcohols, and embedded in paraffin wax. From each paraffin block, 5 µm sections were obtained and stained with the following histochemical routine techniques: Hematoxylin–Eosin and Masson’s trichrome.

### 2.3. Immunohistochemical Technique

The immunohistochemical methodology and commercial kit identification for antibodies are summarized in Table 2.

Briefly, sections immunolabelled with anti-actin (RRID: AB_262054) and anti-von Willebrand factor (RRID: AB_2811207) antibodies were visualized using a Chemmate Dako EnVision detection kit (Glostrup-Denmark). The Peroxidase™/DAB, Rabbit/Mouse System (Dako, Glostrup, Denmark) was used following the manufacturer’s instructions. Sections of human aortas were used as positive controls for actin. Vascularized pheochromocytoma tumors from chromaffin cells of the adrenal medulla with large micro-vessels sections were used as positive controls for the von Willebrand factor. The tissue bank of the Pathologic Anatomy Service from the Complejo Hospitalario Insular Universitario Materno Infantil de Las Palmas de Gran Canaria (Spain) provided the human positive control samples for immunohistochemical positive controls. An antihuman monoclonal anti-actin alfa-smooth muscle antibody produced in mice was used at a 1:2000 dilution for actin immunodetection. For the von Willebrand factor, immunodetection was performed with an antihuman polyclonal anti-factor VIII-related antibody produced in rabbits at a 1:100 dilution. In both cases, endogenous peroxidase was blocked for 30 min with 3% hydrogen peroxide in methanol. The samples were analyzed with a Labophot-2 Nikon microscope. Images were captured with a Sight DS-5M digital camera.

### 2.4. Measurements of the Aortic Wall

The 4× and 10× measurements of the aortic wall were performed in randomly selected fields of one Orcein or one Masson trichrome stained histological section of each case, using a Digital Sight Camera Control Unit coupled to a Labophot-2 Nikon microscope. In addition, four different measurements were performed directly in each histological section using digital camera tools. At low magnification (4×), the thickness of the tunica intima, tunica media, and total wall (IM) were measured. The tunica adventitia was not measured because it was impossible to determine its outer limit in all cases. The tunica media thickness was considered as the perpendicular distance between the innermost and outermost elastic lamina. The thickness was measured at four equidistant points within the same aortic ring section. The mean value of the four measurements was calculated. The elastic laminas’ thickness and the tunica media’s lamellar unit thickness were measured separately at higher magnification (10×). The size of the lamellar unit thickness included smooth muscle cells (smcs), the elastin layer, collagen, and the extracellular matrix. We counted the thickness of the lamellar unit in the transversal section from the adluminal side of a smooth muscle cell to the abluminal side of the elastic layer that limits the luminal side of the following smooth muscle cell. Two of the four measurements were performed in the inner part of the aortic wall and two in the outer. Again, the mean value of the four measurements was calculated. The lamellar unit in the abdominal aorta was measured, avoiding the sizeable longitudinal muscle bundles in the inner part of the tunica media. The number of lamellar units was calculated considering each sample’s total wall thickness and the lamellar unit thickness in each sample.

### 2.5. Statistical Analysis

For statistical purposes, the species were grouped and compared within families as follows: Ziphiidae (*Mesoplodon densirostris*, *Mesoplodon bidens*, *Mesoplodon europeaus*, *Ziphiius cavirostris*), Kogiidae (*Kogia breviceps* and *Kogia Sima*), Physeteridae (*Physeter macrocephalus*), and deep-diving species of the Delphinidae family (*Globicephala macrorhynchus* and *Grampus griseus*). Statistical data analysis was performed by the IBM SPSS Statistics 27 (IBM Corp. Released 2020. IBM SPSS Statistics for Windows, Version 27.0. Armonk, NY, USA, IBM Corp). Categorical variables were expressed as frequencies and percentages, and numerical variables were summarized by mean ± standard deviation (SD). Three means of independent samples were compared using the non-parametric Kruskal–Wallis test due to non-compliance with data normality by using the Shapiro–Wilk test. The results were considered statistically significant if the *p*-value < 0.05.

## 3. Results

### 3.1. Animals

Mean ± SD values of length and weight of the specimens by families are shown in Table 1. The four families’ body lengths and mass were significantly different, *p* < 0.000. The highest values were for the Physeteridae family, followed by the Ziphiidae and the deep-diving species of the Delphinidae family. The smallest size and weight were observed in the Kogiidae family.

### 3.2. Ascending Aorta

The IM thickness ranged between 11043.3 μm for Physeteridae and 1834.2 ± 632.6 μm for the Kogiidae family. Ziphiidae and deep-diving Delphinidae species showed intermediate values (Table 3).

**Figure 1:**
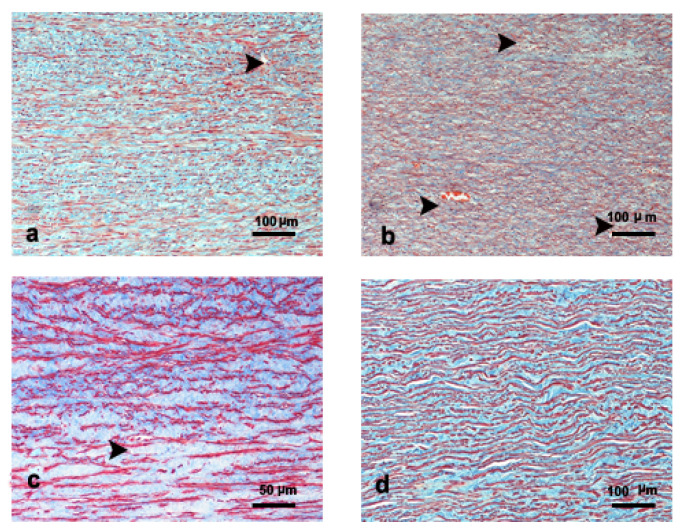
Microscopic findings (trichrome staining) of the ascending aorta in (**a**) *Mesoplodon densirostris* adult male, (**b**) *Globicephala macrorhynchus* adult male, (**c**) *Kogia breviceps* adult male, and (**d**) *Physeter macrocephalus* subadult male. Elastic fibers; dark orange in (**a**,**b**) dark red in (**c**). smcs; light orange in (**a**,**b**), light red in (**c**). Connective tissue; blue in (**c**,**d**), and green-blue in (**a**). Some small vessels are present in the wall (arrowhead). The elastic laminas are located circular and longitudinally.

The ascending aorta presented a thin irregular tunica intima formed by an endothelial cell layer underlined by a thin layer of extracellular matrix. In our samples, the tunica intima thickness was uneven, ranging between 98.7 ± 33.6 μm for Kogiidae and 46.7 μm for Physeteridae, respectively (Table 3). The tunica media comprised elastic laminas, connective matrix, and smcs (Figure 1).

**Figure 2:**
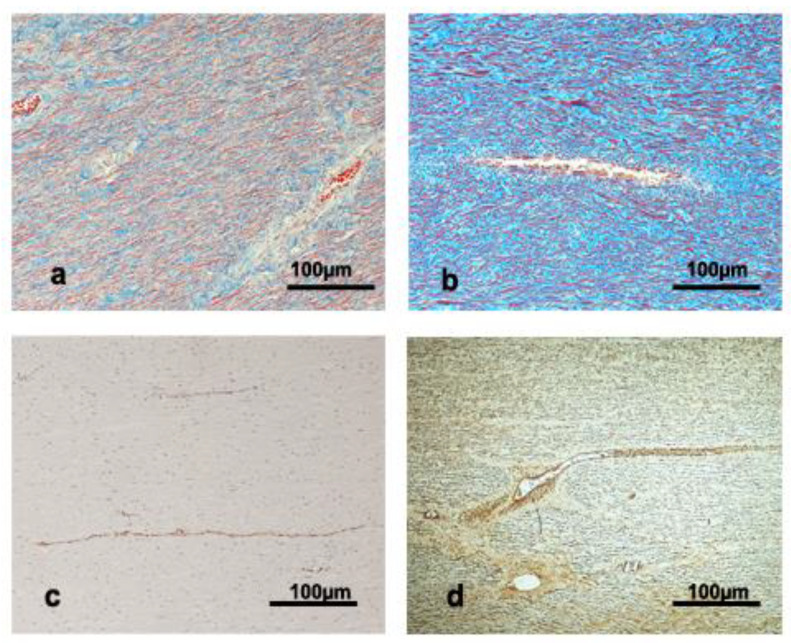
Large blood vessels inside the tunica media, (**a**) thoracic aorta in a calf female *Physeter macrocephalus* (trichromic stain), (**b**) ascending aorta in a subadult male *Physeter macrocephalus* (trichromic stain), (**c**) thoracic aorta in a subadult male *Physeter macrocephalus* (von Willebrand factor immune detection), (**d**) thoracic aorta in a *Mesoplodon bidens* adult female (alfa-actin immunodetection).

The tunica media thickness ranged between 10,996.6 μm for Physeteridae and 1735.5 ± 599.1 μm for the Kogiidae family (Table 3). The elastic lamina alternated circular, longitudinal, and even oblique orientation in the transverse sections, issuing connections between them (Figure 1). The elastic layer thickness ranged between 5.4 ± 1.8 μm for Ziphiidae and 3.8 μm for Physeteridae (Table 3). The smcs and the extracellular matrix were parallel to the elastic lamina. The lamellar units formed by the three elements seemed to be broader in the inner third than in the two outer thirds of the aortic wall (Figure 1). The thickness of the lamellar unit in the ascending aorta ranged between 22.1 ± 4.7 μm and 16.8 ± 4.3 μm for Ziphiidae and Delphinidae, respectively (Table 3). The presence of vasa vasorum in the tunica media was frequent (Figure 1a–c and Figure 2b).

The number of lamellar units ranged from 571 for Physeteridae to 102.4 ± 52.6 for Kogiidae (Table 3). The tunica adventitia was composed of diffuse connective tissue, micro-vessels, and nerves.

### 3.3. Thoracic Aorta

**Figure 3:**
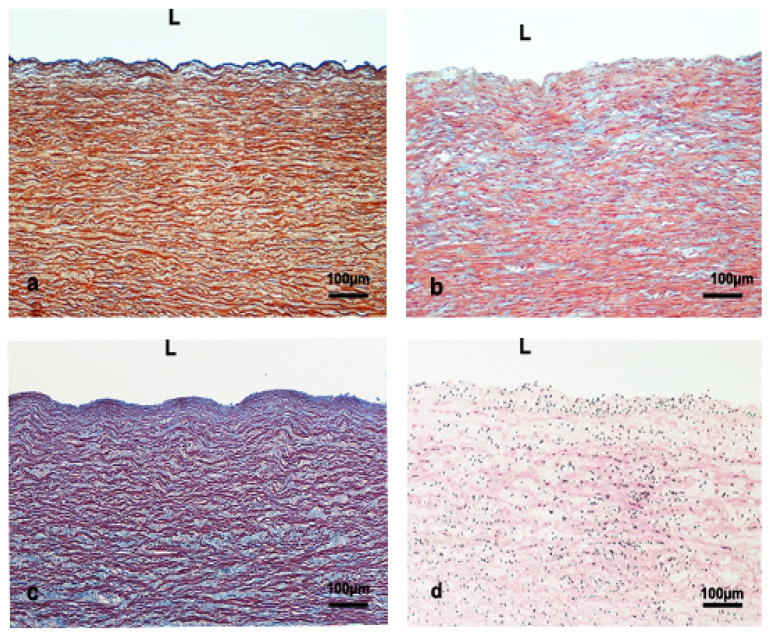
Thoracic aorta sections, (**a**) juvenile male *Grampus griseus* (orcein staining), (**b**) juvenile male *Kogia breviceps* (trichromic staining), (**c**) subadult male *Physeter macrocephalus* (orcein staining), and (**d**) adult male *Mesoplodon densirostris* (hematoxylin/eosin). Elastic fibers; (dark brown) in (**a**) and violet (**c**) and orange in (**b**). Connective tissue: blue in (**b**,**c**), light brown in (**a**). L; lumen. Elastic laminas are mainly circular oriented.

In the thoracic aorta, the IM thickness ranged from 6445.7 ± 2547.3 μm to 1758.5 ± 1170.7 μm for Physeteridae and Kogiidae families, respectively. Delphinidae and Ziphiidae families showed intermediate values (Table 3). The tunica intima ranged between 108.6 ± 107.6 μm and 60.2 ± 41.8 μm for the Kogiidae and Ziphiidae families, respectively (Table 3). No significant morphological differences were observed in the ascending aorta. The tunica media thickness ranged between 6379.9 ± 2573.1 μm and 1679.8 ± 1104.3 μm for Physeteridae and Kogiidae families, respectively (Table 3). Most elastic layers were circularly disposed of in the tunica media, although some were longitudinally oriented. The disposition of the extracellular matrix and smcs were parallel to the elastic layers (Figure 3).

In the thoracic aorta, the thickness of the elastic layers ranged between 6.1 ± 1.5 μm for Physeteridae and 4.1 ± 0.9 μm for Kogiidae. The lamellar unit thickness was wider under the lumen than in the outer part of the tunica media and ranged from 25.5 ± 5.0 μm to 20.05 ± 4.6 μm for Physeteridae and Ziphiidae families, respectively (Table 3). Vasa vasorum was observed deep in the wall in the four families; they approached the internal third of the tunica media in the Kogiidae and Physeteridae families (Figure 2a,c,d). The number of lamellar units ranged from 256.6 ± 121.6 to 72.2 ± 44.4 for Physeteridae and Kogiidae, respectively.

### 3.4. Abdominal Aorta

**Figure 4:**
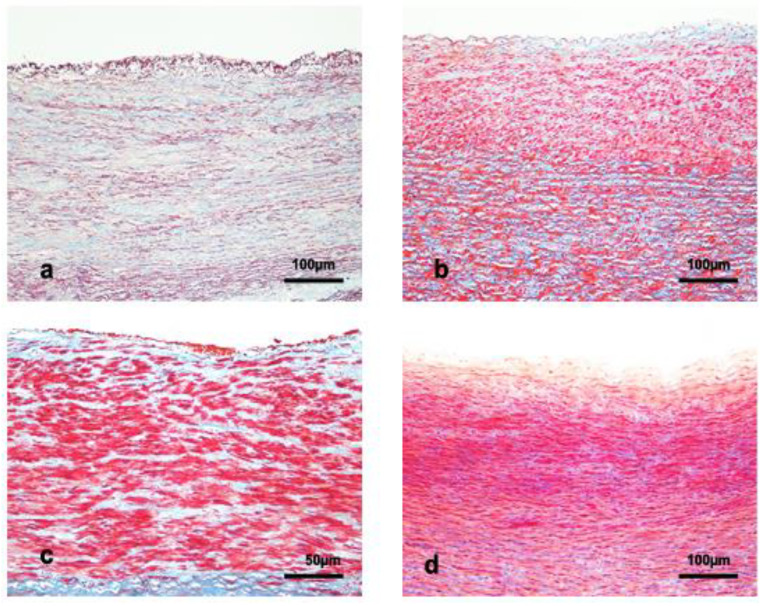
Abdominal aorta section (orcein and trichrome staining) of (**a**) adult female *Mesoplodon densirostris* (orcein staining), (**b**) juvenile female *Kogia breviceps*, (**c**) adult male *Mesoplodon densirostris*, and (**d**) calf female *Grampus griseus*. The red color indicates the smcs in (**b**–**d**), light pink in (**a**); the blue color indicates connective tissue, and the orange-violet color indicates the elastic fibers.

The proximal portion of the abdominal aorta was similar to the thoracic aorta. In the distal portion of the abdominal aorta, the IM thickness ranged between 10,306.6 ± 692.7 μm and 627 ± 292.4 μm for deep-diving Delphinidae and Kogiidae, respectively (Table 3). Regrettably, no abdominal aorta samples were collected from the *Physeter macrocephalus specimens* included in the present work. The tunica intima thickness ranged between 41.8 ± 14.5 μm and 30.0 ± 9.0 μm for Ziphiidae and Kogiidae, respectively (Table 3). This tunica was thin and irregular (Figure 4).

The tunica media thickness ranged from 1269.6 ± 668.5 μm and 597.6 ± 283.4 μm for the deep-diving Delphinidae species and the Kogiidae family, respectively. The tunica media in the distal segment was composed of layers of smcs circularly disposed of and in bundles of smcs longitudinally orientated. Those bundles were shared by strands of thick laminas of connective tissue, appearing as columns in most samples (Figure 4). The elastic layers were thin and fragmented, surrounding the layers and bundles of smcs. The thickness of the elastic layers ranged between 4.7 ± 0.5 μm for Kogiidae and 3.9 ± 1.1 μm for Ziphiidae (Table 3).

The lamellar units were hardly identifiable in this section of the artery formed mainly for smcs and connective strands. The thickness of the lamellar units in the places identifiable ranged between 19.7 ± 1.7 μm and 18.6 ± 2.3 μm for deep-diving Delphinidae species and the Kogiidae family, respectively. The number of lamellar units ranged between 67.1 ± 42.5 for deep-diving Delphinidae species and 31.4 ± 11.4 for Kogiidae (Table 3). Vasa vasorum was not observed inside the tunica media in this section of the artery.

### 3.5. Complete Aortic Wall

**Figure 5:**
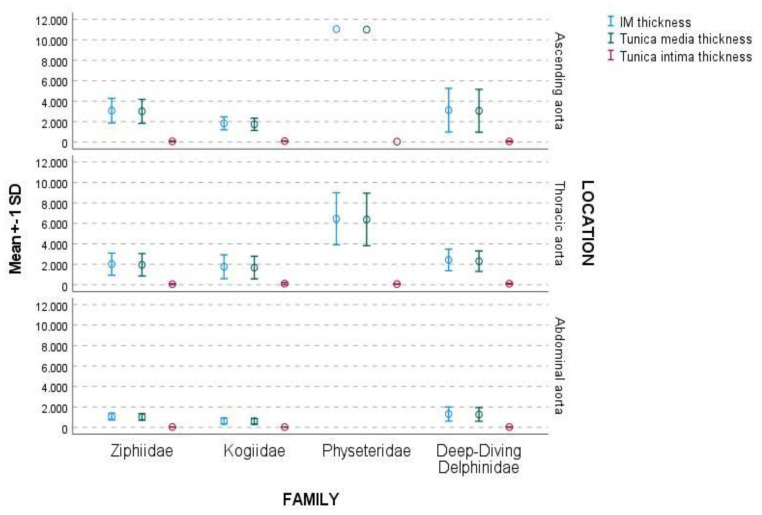
The graphic shows the mean ± SD thickness (μm) of the IM, tunica media, and tunica intima in the four families attending to the aortic location.

Figure 5 exhibits each family’s IM, tunica media, and tunica intima thickness in the three aortic locations: ascending, thoracic, and abdominal aorta.

**Figure 6:**
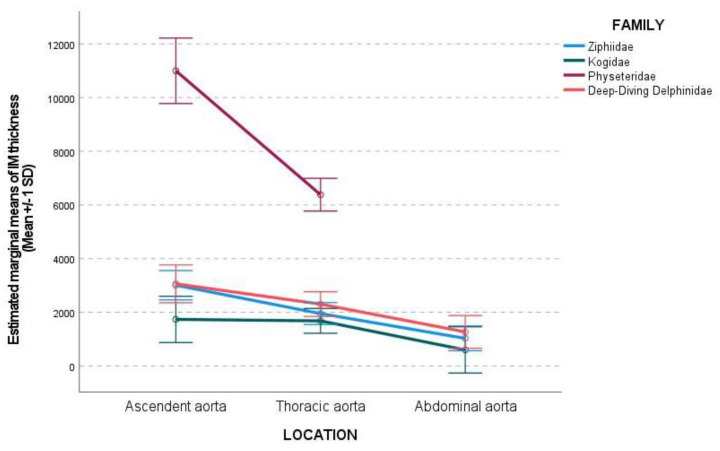
Representation of the aortic wall thickness (μm) at each aortic location by family. A decrease in IM and tunica media thickness was observed between the ascending, thoracic, and abdominal aorta. The difference between ascending and thoracic aorta thickness was higher in the Physeteridae family than in the other families.

The IM and the tunica media thickness were higher in the ascending aorta than in the thoracic aorta and in the thoracic aorta than in the abdominal aorta (Table 3). The difference in thickness in the IM and tunica media between the ascending and thoracic aorta was dramatic in the Physeteridae family (Table 3, Figure 6).

**Figure 7:**
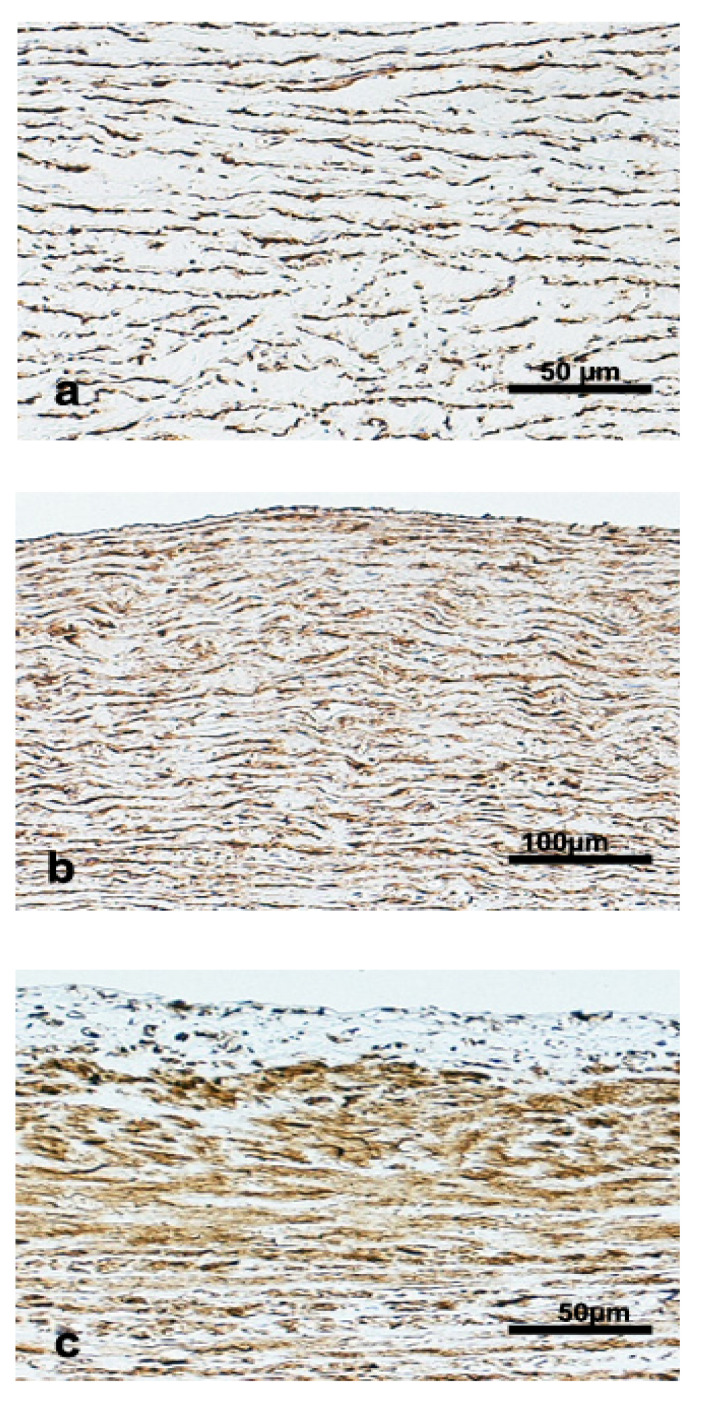
Alfa- actin immunodetection in the (**a**) ascending aorta of a juvenile male *Kogia breviceps*, (**b**) thoracic aorta of an adult male *Globicephala macrorhynchus*, (**c**) abdominal aorta of an adult female *Mesoplodon densirostris*. Brown staining indicates the presence of smcs in the aortic wall.

The IM thickness and tunica media thickness were higher for the Physeteridae family, followed by deep-diving Delphinidae species and the Ziphiidae family, being the lowest values for the Kogiidae family (Table 3). The amount of smcs in the tunica media increased from ascending to the thoracic and abdominal aorta (Figure 7).

The IM and the tunica media thickness in the thoracic aorta concerning the ascending aorta are shown in Table 4.

**Figure 8:**
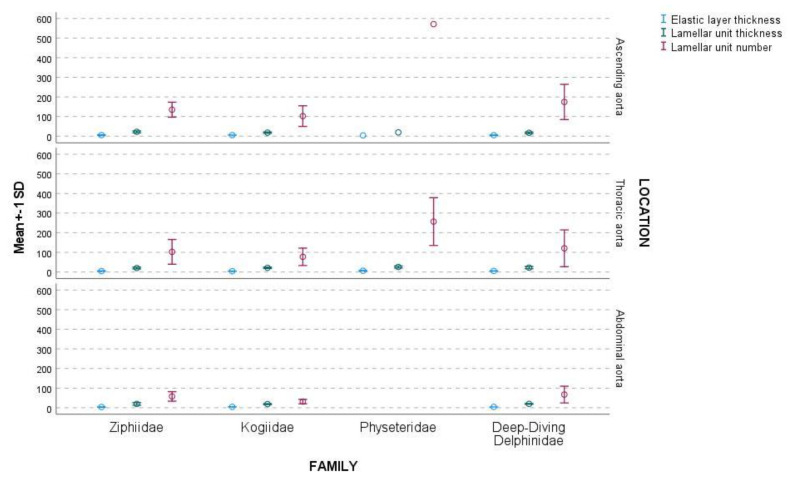
Representation of the mean ± SD (μm) of the elastic and lamellar unit thickness and the number of lamellar units by aortic location and family.

The highest differences were found in the Physeteridae family, followed by the Ziphiidae family, deep-diving Delphinidae species, and the Kogiidae family. The IM and the tunica media thickness in the abdominal aorta related to the ascending aorta were similar between families, although there were no data for the Physeteridae family (Table 4). The elastic layers were observed more abundantly in the proximal segments than in the distal parts of the aorta. In addition, the elastic layer thickness showed irregularity in the different aortic locations and families; generally, they were thinner in the distal than the proximal aortic locations (Table 3, Figure 8).

The mean and SD for elastic layer thickness were 4.8 ± 1.3 µm, and the values for all families ranged between 2.3–8.0 µm. The mean value of the lamellar unit thickness for all families was 20.7 ± 4.7 µm, ranging between 9.7–33.0 µm (Table 5).

The tunica intima’s thickness was irregular, being thinner in the abdominal aorta than in the other aortic locations in all families (Table 3, Figure 5). The thickness of the tunica intima was narrow compared to the rest of the tunics, regardless of the aortic location or family. The mean value was 69.9 ± 55.9 µm, ranging between 8.3–272.5 µm (Table 4).

## 4. Discussion

Cardiovascular adaptations are necessary for allowing marine mammals to dive deep and for long durations. Considering that the differences in the properties of the aorta along its length represent the adaptation to the stresses that the different aortic locations experience [20], this work aimed for the first time to describe the characteristics of the aortic wall of cetacean with deep diving habits belonging to four different families: Ziphiidae, Kogiidae, Physeteridae, Delphinidae, being valid parameters to evaluate the aortic wall characteristics the IM thickness, the lamellar unit thickness and the number of lamellar units in the tunica media. These parameters have been previously considered by several authors studying Fin whales [3,4,9,11], Harbor and Weddel seals [8], ten terrestrial mammal species [21], humans [22], and Landrace pigs [23].

### 4.1. Structure of the Aortic Wall

The structure of the aortic wall of the analyzed species was similar to what has been described previously for shallow and intermediate diving dolphins or other marine mammals such as the northern elephant and harbor seals [11], showing an elastic ascending aorta with elastic layers oriented in all directions. These species also showed a thoracic aorta with elastic layers mostly circularly oriented and an abdominal aorta with bundles of smcs shared by bands of fibrous tissue. As observed in dolphins with shallow and intermediate diving habits and terrestrial mammals, the smcs were scarce in the proximal aortic location and increased from proximal to the distal aorta [23]. In all species, the disposition of the aorta’s structural components corresponded with an elastic ascendant and a more rigid and muscular abdominal aorta.

### 4.2. IM and Tunica Media Thickness

Our results showed that the IM and tunica media thickness varied in the four studied families: from thicker to thinner in the Physeteridae, deep-diving Delphinidae species, Ziphiidae, and Kogiidae families, respectively. Initially, one could think that wall thickness could be related to the animal’s body mass and length. The Physeteridae specimens were the longest and heaviest, while the Kogiidae specimens were the shortest and lightest. Still, the relationship was not coincident with the representatives of the Ziphiidae family and deep-diving Delphinidae species. Ziphiidae specimens showed higher weight and length than the deep-diving Delphinidae species. However, the IM and tunica media thickness were higher in the deep-diving Delphinidae species than in the Ziphiidae family. These last findings reject the size and weight of the animal as the only variable related to the thickness of the aortic wall. Concerning other studied cetaceans, the aortic wall thickness of the Physeteridae specimens showed to be thinner than the aortic wall of the fin whales of the Balaenopteridae family [3,9]. The thickness of the aortic wall of deep-diving Delphinidae species was thicker than those with shallower diving habits within the same family [13]. The IM and tunica media thickness decreased along the aorta in all cases. This is a common characteristic of terrestrial [20,23,24] and marine mammals [3,8,9]. However, the decrease in the IM thickness and the tunica media thickness in the aortic locations along the artery did not have the same ratio for all species. In our study, the tunica media of the abdominal aorta represented 30–42% of the thickness in the ascendant aorta in all families; however, the decrease between the ascending and thoracic aortic locations was very different depending on the family, being more dramatic in the Physeteridae family, in which the thickness in the thoracic aorta was about half of the ascending aorta, similarly to the difference described for the Weddell seal (~50%) [8]. Following the Physeteridae family, a higher reduction was observed in the Ziphiidae family, followed by the deep-diving Delphinidae species. At the same time, the Kogiidae family presented a very mild decrease in the aortic wall thickness.

### 4.3. Lamellar Unit

The lamellar unit is the structural and functional unit of the aorta’s tunica media [25] and is related to aortic compliance [26]. The structure and morphometry of the aortas of dolphins with shallow and intermediate diving habits were similar to the terrestrial mammals except for the more considerable difference in the proportion of lamellar units between ascend and thoracic aorta [13].

We observed in a previous work a reduction in the lamellar unit thickness between the proximal and distal aortic segments in dolphins with shallow and intermediate diving habits. Those findings agreed with the conclusions of the fin whale [9] and in contraposition to what has been described for Landrace pigs [23]. A different measurement system could explain these differences in the abdominal aorta where the bundles of smcs are present.

In the current study in deep-diving cetaceans, we could not observe an increase or decrease in the lamellar unit thickness attending to the aortic location in the four families of cetaceans, and the lamellar unit thickness showed certain irregularity in most cases. The distance between the elastin lamina has been considered relatively uniform along the aortic circumference and depth [21]. The thickness of the lamellar unit was not uniform being slightly wider under the lumen than in the outer part of the vascular wall, and it was unrelated to the aortic location or family studied. The changes in lamellar unit thickness with the distance from the lumen might be related to the artery’s physiological state.

The highest number of lamellar units in the tunica media was found in the Physeteridae family, followed by the deep-diving Delphinidae species and the Ziphiidae and Kogiidae families. The number of lamellar units was closely related to the IM and tunica media thickness in all aortic locations. Its number reduced significantly between the ascending aorta and thoracic aorta. In the current study, the reduction in the lamellar unit number between both locations was higher for Physeteridae samples than for the rest of the families. In the Physeteridae specimens, the number of lamellar units in the thoracic aorta was 45% of those present in the ascending aorta, which implies an essential difference in the compliance between the two aortic segments. Higher differences have been described for the fin whales, with a number of lamellar units in the thoracic aorta equivalent to 20% of the number in the ascendant aorta [9]. The deep-diving Delphinidae species and Kogiidae and Ziphiidae families presented fewer differences between both aortic locations, and they were more similar to what has been described for shallow and intermediate diving dolphins. There were no observed significant differences between the shallow and intermediate deep-diving dolphins, and deep-diving species of dolphins, except for the IM, media thickness, and the number of lamellar units. The similarity could be related to the presence of intermediate deep-diving species in the first study.

The thickness of the elastic layers in the lamellar unit was similar for the four families and aortic location.

### 4.4. Vasa Vasorum

The vasa in the tunica media of the mammals’ aortic wall have been related to pathological conditions [27,28] and the tunica media’s size. It is considered that they are present in a tunica media with more than 29 lamellar units [21] or a tunica media thickness larger than 0.5 mm [29]. We found vasa in the tunica media of the ascending and aortic thoracic aorta. The vasa, on occasions, extended to the internal third of the tunica media without pathological conditions. Vasa was not observed in the tunica media of the abdominal aorta samples, although the tunica media had more than 29 lamellar units [21]. The lack of vasa in the abdominal aorta has also been described in humans [22], and pigs [23].

### 4.5. Aortic Wall and Diving

Considering the diving habits described for Physeteridae [30,31], Kogiidae [32,33], deep-diving Delphinidae species [34], and Ziphiidae [35,36], our findings support that a decrease in the IM and tunica media thickness apart from the loss in lamellar units, between the ascending and thoracic aorta, is the most relevant parameter related to the diving adaptation.

### 4.6. Limitations

Our study had limitations concerning the sample size and the number of specimens for each family. Furthermore, the characteristics of the specimens studied heterogeneously belonged to different species, sex, and age categories, so it was not possible to perform a deeper statistical analysis correlating the different variables. Other limiting factors were the study’s retrospective nature, with missing samples from some aortic locations in some specimens and the postmortem artifacts. The morphometry of the tissue might be modified postmortem (e.g., contracted arteries) and is not directly translational to living organisms. However, comparative analyses under the same conditions should render translational differences in living organisms. Given the ethical, logistical, and technical difficulties of obtaining these measurements from these cryptic and elusive animals, studies on dead-stranded animals with the same decomposition code represent a fair approximation and a first step in understanding the vascular morphological adaptations of marine mammals to dive.

## 5. Conclusions

The findings of this work point out that the decrease in the IM and tunica media thickness and the loss of lamellar units between the ascending and thoracic aorta may be the most representative of aortic wall morphometric and morphological adaptation to diving among the analyzed parameters. The higher thickness and elasticity of the proximal segment would allow more elevated and more sure storage of systolic flow during diving; meanwhile, the thinner and less elastic thoracic aorta would allow a more continuous peripheral circulation of the diastolic flow during diving. Therefore, the difference between these two segments could imply better capacitation for diving.

## Figures and Tables

**Table 1:** Summary of species, sex, life history categories, body length, and mass of the studied cetaceans. M: male, F: female, U: unknown, T: total cases, N: number of samples. * Kruskal–Wallis test.

FAMILY	SPECIES	M	F	U	T	LIFE STAGES	N	LENGHT	WEIGHT
**ZIPHIIDAE**	*Mesoplodon densirostris* (De Blainville, 1817)	**2**	**1**	**1**	**4**	unknown	1	401.6 ± 79.3	645.6 ± 324.4
						young	**1**		
						adult	2		
	*Mesoplodon Europeaus* (Gervais, 1855)			1	1	calf	1		
	*Mesoplodon Bidens* (Sowerby, 1804)		1		1	adult	1		
	*Ziphius cavirostris* (Cuvier, 1823)	**2**	**1**	**1**	**4**	unknown	1		
						young	**2**		
						adult	1		
**PHYSETERIDAE**	*Physeter macrocephalus,* (Linnaeus 1758)	**3**	**1**		**4**	calf	1	776.2 ± 214.6	5375 ± 2750
						young	**1**		
						adult	2		
**KOGIIDAE**	*Kogia breviceps* (De Blainville, 1828)	3	4		7	calf	4	177.4 ± 52.8	107.1 ± 112.6
						young	2		
						adult	1		
	*Kogia Sima*(Owen, 1866)		1		1	calf	1		
**DELPHINIDAE**	*Globicephal macrorhynchus* (Gray, 1846)	3	2		5	young	3	276.8 ± 95	340.1 ± 325.7
						adult	2		
	*Grampus Griseus* (Cuvier, 1812)	1	3		4	calf	3		
						young	1		
	**SUMMARY**	15	15	3	31	unknown	2	*p* = 0.000 *	*p* = 0.000 *
						calf	10		
						young	11		
						adult	10		

**Table 2:** Summary of immunohistochemical staining methodology. (a) 0.1%Tripsina, 60 min at 37° (b) 1% goat serum in saline phosphate buffer, 4 °C overnight. RRID (Research Resource Identifier).

Antibody	Manufacturer	RRID	Host	Type	Clone	Antigen Retrieval	Dilution
Anti- Actin	Sigma–Aldrich, St. Louis, MO, USA Cat# A5228	AB_262054	mouse	Monoclonal	Anti-actin α smooth-muscle antibody-1A4	0.1% trypsin (a)	1:2000 (b)
Anti-Factor VIII	Zymed laboratories Inc, San Francisco CA94080 Cat# 18-0018	AB_86604	rabbit	Polyclonal		0.1% trypsin (a)	1:100 (b)

**Table 3:** Mean and SD, as well as a minimum (Min) and Maximum (Max) values of the IM, tunica media, tunica intima, and elastic thickness, as well as lamellar unit thickness and number by aortic location and families.

ASCENDING AORTA				THORACIC AORTA			ABDOMINAL AORTA		
**IM THICKNESS (μm)**									
**Family**	***n***	**Mean ± SD**	**Min–Max**	***N***	**Mean ± SD**	**Min–Max**	***n***	**Mean ± SD**	**Min–Max**
**Ziphiidae**	5	3079.8 ± 1202.1	1697.5–4919.8	9	2015.4 ± 1082.2	856.5–3837.1	7	1069.5 ±333.8	765.1–1775.8
**Kogiidae**	2	1834.1 ± 632.6	1386.8–2281.5	7	1758.5 ± 1170.7	488.1–3844.3	2	627.6 ± 292.4	420.8– 834
**Physeteridae**	1	11043.3	-	4	6445.7 ± 2547.2	4170.1–9782.6	0	-	-
**Deep-diving Delphinidae**	3	3124.1 ± 2132.5	1119.8–5365.1	7	2426 ± 1057.9	1209.9–4372.9	4	1306.5 ± 692.7	845.1–2330.9
**MEDIA THICKNESS (μm)**									
**Ziphiidae**	5	3008.8 ± 1173.3	1643.3–4790.3	9	1951.7 ± 1094.8	829.6–3763.5	7	1027.7 ± 322.9	733.3–1716.4
**Kogiidae**	2	1735.4 ± 599.1	1311.8–2159.1	7	1679.7 ± 1104.3	441.4–3571.8	2	597.6 ± 283.4	397.2–798
**Physeteridae**	1	10996.5	-	4	6379.8 ± 2573.1	4097.4–9751.2	0	-	-
**Deep-diving Delphinidae**	3	3059.5 ± 2099.8	1108.5–5281.8	7	2303.8 ± 999.6	1133.7–4151.1	4	1269.6 ± 668.4	815.7–2258,2
**INTIMA THICKNESS (μm)**									
**Ziphiidae**	5	71 ± 40.2	19.4–129.4	9	60.1 ± 41.8	8.2–147.8	7	41.8 ± 14.4	28.6–62.5
**Kogiidae**	2	98.7 ± 33.5	74.9–122.4	7	108.6 ± 107.5	31.8–272.4	2	29.9 ± 8.9	23.6–36.3
**Physeteridae**	1	46.7	-	4	66.1 ± 31.1	28.9–102.6	0	-	-
**Deep-diving Delphinidae**	3	64.5 ± 46.7	11.2–99	7	100.4 ± 59.8	46.8–221.8	4	36.9 ± 26.1	10.3–72.7
**ELASTIC THICKNESS (μm)**									
**Ziphiidae**	5	5.4 ± 1.7	3.2–7.9	9	4.5 ± 1.4	3.1–7.2	7	3.9 ± 1.1	2.26–5
**Kogiidae**	2	5.2 ± 0.3	4.9–5.5	7	4.1± 0.8	2.6–5.3	2	4.7 ± 0.4	4.4–5
**Physeteridae**	1	3.7	-	4	6.1 ± 1.4	4.5–7.9	0	-	-
**Deep-diving Delphinidae**	3	5.1 ± 0.9	4.1–6.1	7	5.4 ± 0.9	4.1–6.8	4	4.3 ± 0.2	4–4.6
**LAMELLAR UNIT THICKNESS (μm)**									
**Ziphiidae**	5	22 ± 4.7	16.8–26.9	9	20,05 ± 4.5	13.8–27.3	7	19.4 ± 6.4	9.7–26.2
**Kogiidae**	2	17.8 ± 3.3	15.4–20.1	7	21,3 ± 3.1	16.5–25.3	2	18.6 ± 2.2	17–20.2
**Physeteridae**	1	19.2	-	4	25. 5 ± 5	22.3–32.9	0	-	-
**Deep-diving Delphinidae**	3	16.7 ± 4.2	13.5–21.6	7	22.1 ± 5.7	13.1–27.5	4	19.69 ± 1.6	17.4–20.9
**NUMBER OF LAMELLAR UNITS**									
**Ziphiidae**	5	135 ± 38.3	97.5–182.4	9	102.4 ± 62.9	38–224.7	7	57.9 ± 24.3	36.8–104.2
**Kogiidae**	2	102.3 ± 52.5	65.1–139.5	7	72.2 ± 44,4	21.3–140.7	2	31.4 ± 11.3	23.3–39.4
**Physeteridae**	1	570.9	-	4	256.6 ± 121.6	169.7–436.7	0	-	-
**Deep-diving Delphinidae**	3	174.3 ± 89.8	73–244.2	7	120.6 ± 93.8	58.2–317.4	4	67.1 ± 42.4	39.1–129.9

**Table 4:** Relationship of the IM and tunica media thickness and the number of lamellar units between the aortic location by families.

IM THICKNESS	Thoracic/Ascending Aorta	Abdominal/Ascending Aorta
Ziphiidae	65%	34.7%
Kogiidae	95%	34.2%
Physeteridae	58.3%	-
Deep-diving Delphinidae	77.6%	41.8%
**MEDIA THICKNESS**	Thoracic/ascending aorta	Abdominal/Ascending aorta
Ziphiidae	64%	34,1%
Kogiidae	96.7%	34.4%
Physeteridae	58%	
Deep-diving Delphinidae	75.3%	41.4%
**LAMELLAR UNITS**	Thoracic/ascending aorta	Abdominal/Ascending aorta
Ziphiidae	75.8	42.8%
Kogiidae	70.5%	30.6%
Physeteridae	44.9%	-
Deep-diving Delphinidae	62.2%	39.6%

**Table 5:** Mean ± SD and maximum and minimum values in all the families.

	*N*	Mean ± SD	Minimun–Maximun
Intima thickness μm	51	69.8 ± 55.9	8.2–272.4
Elastic thickness μm	51	4.7 ± 1.2	2.2–7.9
Lamellar unit thickness μm	51	20.6 ± 4.7	9.7–32.9

## Data Availability

Data are available upon reasonable request.
